# Quantifying the Activation Barrier for Phospholipid Monolayer Fusion Governing Lipid Droplet Coalescence

**DOI:** 10.3390/ijms262311664

**Published:** 2025-12-02

**Authors:** Rodion J. Molotkovsky, Zaret G. Denieva, Ivan N. Senchikhin, Ekaterina K. Urodkova, Petr V. Konarev, Georgy S. Peters, Timur R. Galimzyanov, Rais V. Pavlov, Pavel V. Bashkirov

**Affiliations:** 1 Research Institute for Systems Biology and Medicine (RISBM), Nauchnyi Proezd 18, 117246 Moscow, Russia; 2 A.N. Frumkin Institute of Physical Chemistry and Electrochemistry, Russian Academy of Sciences, 119071 Moscow, Russia; 3 National Research Centre “Kurchatov Institute”, Akademika Kurchatova pl. 1, 123182 Moscow, Russia; 4 Independent Researcher, 80634 Munich, Germany

**Keywords:** lipid droplets, lipid membrane, monolayer fusion, dynamic light scattering, theory of lipid membrane elasticity

## Abstract

Lipid droplet (LD) coalescence is a critical cellular process that reshapes lipid storage, drives metabolic disease progression, and dictates the stability of LD-mimetic drug carriers. However, the rate-limiting step—fusion of the phospholipid monolayers surrounding neutral-lipid cores—remains poorly quantified compared to bilayer fusion. Here, we quantitatively determine the activation barrier for LD coalescence by tracking the kinetics in protein-free adiposome models. Using a multi-technique approach combining time-resolved dynamic light scattering and small-angle X-ray scattering, we reveal that monolayer fusion is the kinetic bottleneck. We demonstrate that lipid composition is a powerful regulator of this barrier: cone-shaped lipids (e.g., dioleoylphosphatidylethanolamine) lower the barrier and promote fusion, while phosphatidylcholine-rich monolayers enhance stability. A continuum fusion model, adapted for curved monolayers, explains these results through changes in spontaneous curvature, hydration repulsion, and stalk energetics. Our findings establish composition-dependent design rules for controlling LD dynamics in metabolic health and for engineering stable or triggerable lipid-based delivery vehicles.

## 1. Introduction

Lipid droplets (LDs) are ubiquitous cellular organelles responsible for the storage and transport of neutral lipids. Their unique architecture—a neutral lipid core enclosed by a phospholipid monolayer, primarily composed of lipids like DOPC (dioleoylphosphatidylcholine) and DOPE (dioleoylphosphatidylethanolamine) [1]—defines their interfacial mechanics, stability, and interactions [2]. Within cells, LD size is dynamically regulated by a network of processes. Enzymes on the LD surface, such as lipases and acyltransferases, directly modulate size by catalyzing triglyceride hydrolysis (lipolysis) or synthesis (lipogenesis), thereby shrinking or expanding the hydrophobic core [3]. Alongside this enzymatic regulation, LD size is also controlled by their merging, a physical remodeling event involving fusion of the lipid-monolayer shells followed by union of the neutral lipid cores of adjacent LDs [2,3,4]. This merging can be protein-mediated; for example, Cide family proteins assemble fusion pores between adjacent LD monolayers to enable direct neutral lipids transfer in adipose tissue [5]. This transfer was observed by direct experiments measuring the sizes of merging droplets during their fusion [6] and is a consequence of the difference in Laplace pressures in droplets of different sizes. Yet, protein-free LD fusion—spontaneous coalescence of both shells and cores—has also been observed in vitro and in vivo, underscoring the intrinsic physicochemical instability of the LD monolayer [7,8]. In this protein-free regime, monolayer composition acts as a key regulatory switch: increasing the fraction of cone-shaped DOPE promotes larger LDs, whereas increasing the cylindrical DOPC enhances stability and reduces mean size [7,9]. Consequently, LDs can be viewed as metastable emulsions whose fate is governed by the biophysical properties of their interfacial monolayer [2].

The physiological consequences of uncontrolled monolayer-driven coalescence are profound. Aberrant LD growth is linked to metabolic pathologies such as hepatic steatosis and obesity, where altered LD size distribution disrupts cellular function [10,11]. This fundamental stability challenge extends directly to biotechnology: synthetic lipid nanoemulsions engineered as LD-mimetic drug delivery vehicles [12,13] face the same interfacial constraints; their storage stability and controllable release profiles are dictated by the fusogenicity of their phospholipid monolayers [14,15]. This creates a design paradox: maximizing stability for storage conflicts with enabling rapid content release at the target site. A quantitative understanding of monolayer fusion is therefore essential both for unraveling metabolic disease mechanisms and for rationally engineering LD-like delivery systems with programmable stability and release characteristics [16].

Despite this importance, the rate-limiting interfacial step of LD coalescence—fusion of phospholipid monolayers around neutral-lipid cores—remains far less quantified than bilayer fusion [17,18]. Established emulsion theory posits that flocculation (aggregation) and coalescence drive the time-dependent decline in particle number [19]. For submicrometer LD mimetics, flocculation is typically rapid and reversible [20], placing the kinetic bottleneck on the activation barrier for monolayer fusion upon contact. This process involves a transition state characterized by local monolayer deformation, hydrophobic defect formation, and a stalk/inverted-pore configuration [21,22]. The barrier is exquisitely sensitive to lipid molecular shape, quantified by the so-called spontaneous curvature: surfactants whose spontaneous curvature better matches the transition-state geometry lower the deformation energy and accelerate coalescence [22,23]. This framework predicts that cone-shaped lipids like DOPE should facilitate fusion by reducing the barrier [24], a notion supported qualitatively but lacking rigorous experimental quantification.

Here, we address this gap by quantifying, to our knowledge, the first experimental quantification of the activation barrier for phospholipid-monolayer fusion in protein-free LD coalescence. Using protein-free submicrometer adiposomes with a triolein core and a defined phospholipid monolayer [25], we combined time-resolved dynamic light scattering (DLS) with small-angle X-ray scattering (SAXS). Arrhenius analysis of early-stage coalescence kinetics across temperatures yielded the composition-dependent activation barriers. Furthermore, we refined a continuum fusion model to account for the finite curvature of merging adiposomes, enabling a quantitative reconciliation of the observed DOPE-dependent barrier reduction with the theoretical roles of spontaneous curvature, hydration repulsion, and stalk energetics [24]. Together, our results establish definitive, composition-based design rules for controlling LD stability, with direct relevance to metabolic diseases and the engineering of advanced lipid-based delivery systems.

## 2. Results

### 2.1. Colloidal Characteristics of Lipid Droplets

**Figure 1:**
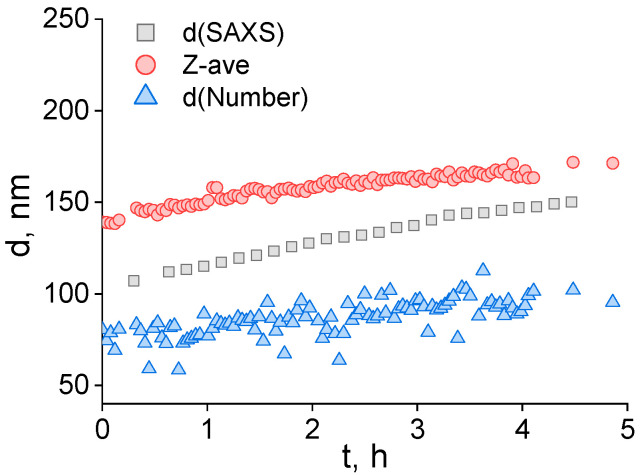
Time evolution of lipid droplet diameter for monolayers containing 35 mol% DOPE at 35 °C. Shown are the number-weighted mean from DLS (blue triangles), the cumulants Z-average hydrodynamic diameter from DLS (pink circles), and the SAXS-derived equivalent spherical diameter (gray squares; *d* = 2*R* with *R* = 1.29*R_g_*).

We monitored adiposome stability and growth kinetics by DLS, collecting number-weighted means, intensity-distribution means, and the cumulants-based Z-average for emulsions containing 0, 20, 35, and 50 mol% DOPE in a DOPC-based monolayer (Table 1). Immediately after preparation, suspensions displayed narrow, largely monomodal size distributions with polydispersity indices (PdI) of 0.14–0.27 and initial number-weighted diameters clustered at ~55–77 nm. As expected, intensity-distribution means were larger due to disproportionate scattering from rare, bigger particles. To select a single kinetic descriptor, we benchmarked the DLS observables against SAXS for the 35 mol% DOPE sample at 35 °C. Time-resolved SAXS (Figure S1) yielded a radius of gyration *R_g_* that increased with time; converting to an equivalent spherical radius (*R* = 1.29*R_g_*) produced a diameter trajectory *d* = 2*R* that lay between the DLS number-weighted mean and the Z-average (Figure 1). Because Guinier-derived *R_g_* underestimates particle size in polydisperse systems, the converted SAXS diameter aligning closest to the Z-average indicates that the cumulants Z-average best tracks the SAXS trend, whereas the number-weighted mean and the intensity-distribution mean systematically underestimate and overestimate size, respectively. Accordingly, we used the Z-average as the primary metric for the characteristic droplet diameter *d* in kinetic analysis.

**Figure 2:**
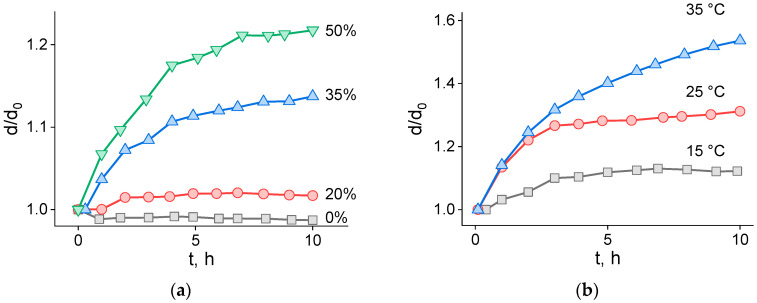
The effects of DOPE content at 30 °C (**a**) and temperature (**b**) on the kinetics of lipid droplet growth (50% DOPE lipid droplets).

For each LD monolayer composition, we recorded the temporal evolution of the characteristic diameter *d*(*t*) across a range of temperatures. Figure 2 compiles representative families of curves with either temperature (Figure 2a) or composition (Figure 2b) held fixed. LDs whose lipid envelope was composed of pure DOPC remained essentially unchanged for *t* > 30 h, indicating high stability under the applied conditions. In contrast, the 35 and 50 mol% DOPE samples exhibited a pronounced early-time increase in mean diameter over the first few hours, followed by slower growth over the subsequent ~10 h. The LDs containing 20 mol% DOPE in the lipid monolayer behaved as a borderline case—stable near room temperature but demonstrating slow destabilization upon incubation at 50 °C. We excluded these results from the barrier-difference analysis since, at this DOPE content, the barrier height is still sufficiently high to make any coalescence undetectable within the experimental timeframe and conditions. Increasing either the DOPE fraction or the temperature accelerated droplet relative growth, as evidenced by larger *d*(*t*)/*d*_0_ at comparable times.

### 2.2. Coalescence Rate of Lipid Droplets and Coalescence Energy Barriers

For adiposomes with 35 and 50 mol% DOPE in a DOPC-based lipid monolayer, we quantified coalescence kinetics. Following the standard emulsion framework, we assumed that reversible flocculation with rate constant *a* and irreversible coalescence with rate constant *K* occur concurrently, jointly decreasing the particle number *n*(*t*) from its initial value *n*_0_ [19]. We did not separately consider Ostwald ripening, as it is indistinguishable from the flocculation in terms of concentration, because it has the same second-order kinetics [26]. In the coalescence-limited regime *K*/(*an*_0_) << 1, an explicit expression for *n*(*t*) can be derived:

(1) $$\frac{n}{n_{0}}=\exp\left(-Kt\right).$$

**Figure 3:**
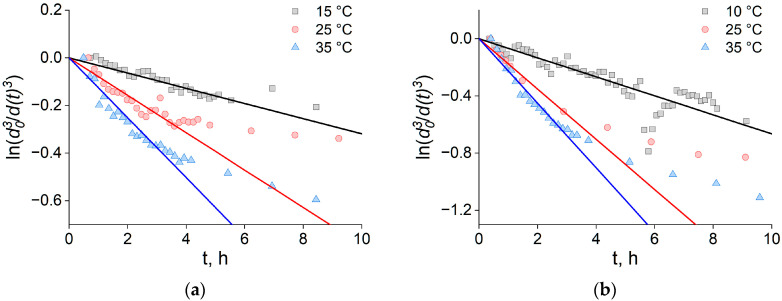
Time dependence of ln(*d*_0_^3^/*d*(*t*)^3^) for lipid droplets at different temperatures (35 °C blue, 25 °C pink, 10 °C gray) measured for monolayers containing (**a**) 35 mol% DOPE and (**b**) 50 mol% DOPE. Symbols denote values derived from DLS Z-average diameters; solid lines are linear fits to the initial coalescence-limited regime used to extract *K*.

This expression is used to analyze the initial-stage kinetics, where the coalescence step is rate-determining [19]. In deriving Equation (1), the coalescence rate is taken to be proportional to the number of thin films formed between contacting particles. To connect *n*(*t*) to experimentally accessible sizes, we used conservation of dispersed volume, which yields:

(2) $$n\left(t\right)\cdot d{\left(t\right)}^{3}=const,$$

so that *n*/*n*_0_ = *d*_0_^3^/*d*(*t*)^3^, where *d*_0_ is the characteristic droplet diameter at *t* = 0. Accordingly, *K* was determined from the slope of ln(*d*_0_^3^/*d*(*t*)^3^) versus time in the earliest linear regime. For each time series, we constructed ln(*d*_0_^3^/*d*(*t*)^3^), averaged the first few points to estimate *d*_0_ robustly, smoothed *d*(*t*) by four-point averaging, and fit the initial interval exhibiting linear behavior. The endpoint of the linear regime was chosen by maximizing the quality of the linear fit (R^2^); the cutoff selection procedure is detailed in the Supplementary Materials (see Figure S2). Representative results for 35 and 50 mol% DOPE are shown in Figure 3a and Figure 3b, respectively.

The concentration decay exhibits a two-stage pattern. During the first ~3 h, ln(*d*_0_^3^/*d*(*t*)^3^) varies linearly with time, indicating a coalescence-limited regime (with coalescence time, *τ_C_* = *K*^–1^ and *K*/(*an*_0_) << 1). At later times, deviations from linearity emerge, consistent with a reduced collision frequency as *n*(*t*) decreases and with flocculation no longer being negligible [19]—behavior also observed in related systems such as Pickering emulsions [20]. In this late regime, accurate descriptions require adding flocculation contributions to *n*(*t*)/*n*_0_ as a sum [27] or series [19]. Because our goal is to extract the coalescence rate, we restricted analysis to the initial linear segment. Coalescence constants *K* with confidence intervals and R^2^ values are reported in Table S1.

We next determined the activation barrier *E* for monolayer fusion using an Arrhenius analysis of the coalescence time:

(3) $$\tau_{C}=\tau^{*}\exp\left(\frac{E}{k_{B}T}\right)$$

**Figure 4:**
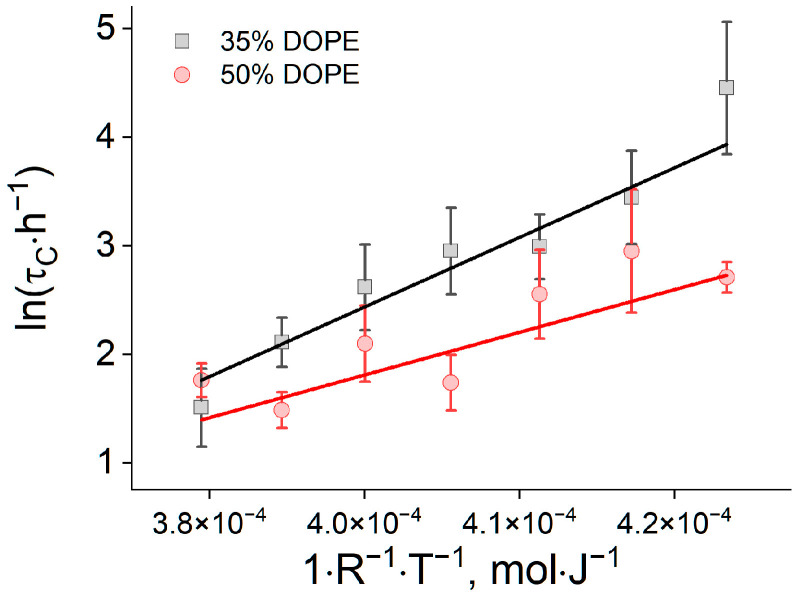
Plots of ln(τ*_C_*) versus (1/*RT*) for 35% DOPE (black) and 50% DOPE (red).

Here, τ* is pre-exponential factor, *k_B_* is Boltzmann’s constant, and *T* is the absolute temperature. Plotting ln(τ*_C_*) versus (1/*RT*) for each composition (Figure 4) and fitting the data linearly yielded barrier heights of *E* = 53.3 ± 7.5 kJ/mol for 35 mol% DOPE and *E* = 32.4 ± 5 kJ/mol for 50mol% DOPE. Our measurements therefore demonstrate that a higher DOPE proportion in the phospholipid monolayer reduces the activation barrier of monolayer fusion by Δ*E* ≈ 20 kJ/mol, which is in a good agreement with the observed coalescence acceleration (Figure 2a).

### 2.3. Theoretical Modeling

The measured composition dependence of the barrier height *E* can be rationalized within a theoretical model of monolayer–monolayer fusion. We generalized our previously developed planar-monolayer fusion model [24] to curved monolayers by considering the fusion of two spherical monolayer shells with identical radius of curvature *R_c_* and a fixed initial distance between the vesicle vertices *h*_0_ (Figure S3). Fusion proceeds through local monolayer deformation that creates a tight contact—the stalk [21,22,24]. Stalk formation is facilitated by the emergence of two symmetrically positioned local hydrophobic defects whose mutual attraction enables necking. The system must cross an activation barrier associated with bending deformation and short-range hydration repulsion [28]. Before stalk nucleation, the free energy is parameterized by the defect radius ρ and the distance *d* between them; after nucleation, the reaction coordinate ρ is continued by redefining it as the stalk radius, providing a single, continuous coordinate across the entire fusion pathway. This construction yields a continuous fusion trajectory and permits calculation of the barrier height *E*. The energy barrier is modulated by changes in the monolayer’s composition, which affect its elastic properties and hydration repulsion. A key mechanism is the change in spontaneous monolayer curvature *J_s_* upon the addition of DOPE. During our analysis, we assumed that the parameters determining the energy of the process depend little on temperature. In particular, we considered a weak temperature dependence of monolayer spontaneous curvature [29] and effective bending modulus [30]. This allowed us to take averaged values of these parameters, corresponding to 300K. Computational details of the energy path construction are provided in the Supplementary Materials, particularly, elastic and geometrical parameters are listed in Table S2.

**Figure 5:**
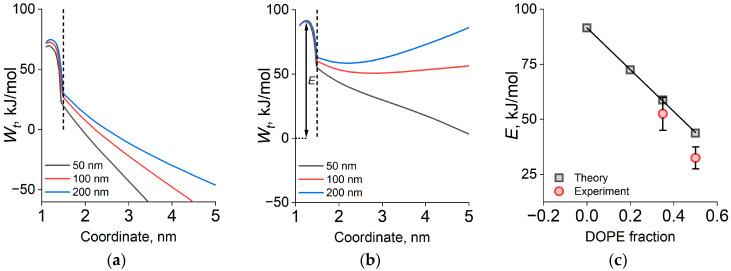
Fusion free-energy trajectories for monolayers with 0 mol% DOPE (**a**) and 35 mol% DOPE (**b**), computed for droplet radii *R_c_* = 200 nm (blue), 100 nm (red), and 50 nm (black). The abscissa is the reaction coordinate ρ; the vertical dashed line marks where *ρ* is redefined from the defect radius (pre-stalk) to the stalk radius (post-stalk). The stalk-formation barrier is denoted *E*. (**c**) Dependence of *E* on DOPE fraction for *R_c_* = 100 nm from theory (gray) and from DLS-based kinetics (red). The solid line provides a visual approximation of the overall trend.

We analyzed the energetics of monolayer fusion for the same compositions that were used to measure the coalesence rates considering LD radii of *R_c_* = 50, 100, and 200 nm, as this radii range is representative of lipid droplet size in vivo [2] and in our measurements (Table 1). Figure 5 shows the calculated fusion trajectories, presented with both pre-stalk (defect convergence) and post-stalk (stalk expansion) branches on a common axis (reaction coordinate) for clarity.

For pure DOPC, our calculations predict that the stalk-formation barrier *E* is essentially independent of curvature, with *E* ≈ 91.5 kJ/mol. Notably, once the stalk is formed, its spontaneous expansion leading to LD coalescence is energetically favorable only for strongly curved shells (*R_c_* ≈ 50 nm); for larger droplets (*R_c_* ≥ 100–200 nm), further increase in stalk radius raises the free energy and thus disfavors expansion (Figure 5a). Computing the corresponding energy trajectories for monolayers containing DOPE revealed a pronounced reduction in *E* with increasing DOPE fraction (Figure 5c). A dependence of *E* on LD curvature was also observed, but it was much weaker than the effect of lipid composition. Importantly, incorporation of approximately 20 mol% DOPE already makes stalk expansion energetically favorable across the entire *R_c_* range examined, thereby converting coalescence into a single-barrier process governed solely by stalk nucleation (Figure 5b). This theoretical outcome is consistent with, and supports, the kinetic model used to extract the activation barrier from the DLS-based coalescence measurements (Figure 4). Crucially, the theoretical barriers align with the kinetic measurements: for both 35 and 50 mol% DOPE, the calculated and experimentally determined *E* values agree well with each other (Figure 4 and Figure 5c). This agreement—both in absolute barrier heights and in the composition-driven decrease in *E*—supports the validity of the kinetic analysis and confirms that lipid composition, rather than curvature, is the primary control parameter for monolayer-fusion energetics over the droplet sizes studied.

## 3. Discussion

This work provides, to our knowledge, the first experimental quantification of the activation energy for phospholipid monolayer fusion that governs protein-free lipid droplet (LD) coalescence. Assuming that early, post-preparation LD growth is controlled by an irreversible, rate-limiting fusion of the apposed phospholipid monolayers, while collisions (flocculation) are rapid and reversible, we measured the temperature dependence of the LD enlargement rate constant, *K*(*T*) and extracted the activation energy *E* via Arrhenius analysis. This approach allowed us to quantify how enrichment of the monolayer with the fusogenic lipid DOPE lowers the monolayer-fusion barrier. We measured *E* ≈ 53.3 ± 7.5 kJ/mol at 35 mol% DOPE and *E* ≈ 32.4 ± 5 kJ/mol at 50 mol% DOPE, indicating a reduction by Δ*E* ≈ 20 kJ/mol upon increasing DOPE by 15 mol% (Figure 4). These barrier magnitudes and their composition dependence align with classical interfacial fusion theory, in which cone-shaped lipids whose spontaneous curvature better matches the stalk/inverted-pore transition state reduce the deformation and hydration penalties and thus accelerate coalescence [22]. Framed within LD biology, the data provide a quantitative basis for earlier observations that phosphatidylethanolamine (PE) enrichment promotes larger LDs and destabilizes LD dispersions, whereas PC-rich monolayers remain resistant to coalescence [7,9,10] and support the view of LDs as metastable emulsions governed by interfacial monolayer physics [2,3]. In particular, the study in [7] records a 5% change in the DOPE fraction, which leads to a 7.25-fold increase in the recorded fusion events. This corresponds to a decrease in the barrier height by 5 kJ/mol, which is quantitatively consistent with our data predicting a decrease in the barrier height by 6.3 kJ/mol with this change in composition, assuming linearity between the decrease in the energy barrier height and the change in the DOPE fraction. Another experimental confirmation of this pattern is the relationship between the proportion of DOPC and the size of lipid droplets in hepatocytes. According to the work in [31], the appearance of large (greater than 1 μm) lipid droplets correlates with an increase in the DOPE/DOPC ratio by approximately 5%. Both of these examples indicate that the experimentally observed changes in the size distribution of lipid droplets are caused by a relatively small shift in the proportion of DOPE. This is in good agreement with the kinetic model we use, where the change in composition is related to the fusion efficiency by an exponential relationship.

To rationalize these trends, we extended a curvature-aware continuum model to compute the free energy trajectory of monolayer fusion along a single reaction coordinate [24] on spherical monolayers, from hydrophobic defect nucleation through stalk formation and expansion to merger, and used it to interpret how DOPE reshapes the fusion free energy landscape. The model predicts that the fusion barrier is far more sensitive to the fraction of conical lipids (e.g., DOPE) than to monolayer curvature across the experimental LD size range and that adding DOPE to DOPC-based monolayers converts fusion into a single-barrier pathway. Mechanistically, a shift in spontaneous curvature of the fusing monolayers toward the negative values favored by the stalk geometry, together with a reduction in short-range hydration repulsion at small intermonolayer distances, lowers the corresponding free energy penalties [18,32,33] and accounts for the composition trend. This same composition dependence governs stalk expansion: for pure DOPC, crossing the initial barrier does not guarantee merger, and droplets can remain metastable (Figure 5), whereas for DOPE fractions ≥ ~20%, expansion proceeds downhill or with a negligible secondary barrier. A simple toroidal-pore picture rationalizes this behavior: for an intermonolayer spacing of ≈ 5 nm and monolayer spontaneous curvature *J_s_* ≤ −0.2 nm^−1^, expansion does not increase the free energy because the geometry of the intermediate matches the intrinsic curvature of monolayers; reported values of *J_s_* ≈ −0.4 nm^−1^ for DOPE and ≈ −0.09 nm^−1^ for DOPC [29] imply that linear mixing of these components to the weighted average *J_s_* ≈ −0.2 nm^−1^ (~35 mol% DOPE) is sufficient to facilitate stalk spontaneous expansion, consistent with our observations (Figure 2). The assumption of linearity of spontaneous curvature in composition is justified by the identity of the hydrocarbon tails of DOPE and DOPC, which ensures that these lipids mix well [29].

Our experimental barriers fall within the 25–87 kJ/mol range reported for water-in-oil and oil-in-water emulsions stabilized by non-lipid surfactants [22,34,35,36]. For example, the coalescence barrier in surfactant-stabilized water/oil/water double emulsions is *E* ≈ 75.5 kJ/mol [35], and water-in-oil emulsions with Span 80 in the presence of Tween 20 exhibit *E* ≈ 85 kJ/mol [21]. Coarse-grained simulations yield barriers near *E* ≈ 25 kJ/mol, with a strong dependence on surfactant geometry [36]. For 20 mol% DOPE samples, we did not observe significant coalescence, while our model predicts *E* ≈ 75.5 kJ/mol, a value that—as per the emulsion literature—can be surmounted by thermal fluctuations over our experimental timescales. This apparent discrepancy is consistent with the differences in the pre-exponential factor, τ*^−1^ (attempt frequency) in Equation (3) across systems: lipid stalk formation is a collective event requiring coordinated remodeling and lipid recruitment, likely lowering τ*^−1^ relative to small-molecule surfactant rearrangements and thereby reducing the observed rate at comparable *E*. Sensitivity to molecular shape in water-in-oil emulsions [21] is also instructive: aqueous-phase coalescence proceeds via pore-like intermediates within the surfactant layer—a pathway topologically distinct from stalk formation—so surfactants that favor positive curvature reduce the coalescence barrier.

Beyond the monolayer bending rigidity *k* and the spontaneous (intrinsic) curvature of the lipid components—which are relatively well characterized and measurable—the activation energy *E* is also strongly governed by the Gaussian curvature modulus κ¯ and by short-range hydration repulsion parameters, both of which are less well constrained because their direct measurement is challenging. A theoretical estimate for a pure DOPC monolayer relates κ¯ to *k* as κ¯ ≈ −0.33·*k* [37]. We adopted this ratio in our calculations of the fusion-barrier height and accounted for the composition dependence of the hydration-repulsion amplitude and decay length by linear interpolation of published values ([38]; see Supplementary Information). As a result, the theoretically calculated barrier height for 50% DOPE turns out to be slightly higher than the experimentally measured value (see Figure 5c). The remaining discrepancy between measured and calculated *E* can be rationalized if the magnitude of κ¯ is overestimated for DOPE-rich monolayers. Indeed, close agreement between theory and experiment is obtained when κ¯ ≈ −0.25·*k* is used for a pure DOPE monolayer, which is physically reasonable [37]. More generally, inserting the measured *E* into our model provides a practical route to infer κ¯, offering an indirect experimental handle on the Gaussian bending modulus.

Comparing LD monolayer fusion to the more ubiquitous and better-studied bilayer fusion reveals two central distinctions. First, monolayer fusion is effectively a single-barrier process, whereas bilayer fusion typically proceeds through two to three sequential barriers before full content mixing [14]. Second, LD monolayer fusion does not require pronounced chain-tilt deformations during stalk formation because no hydrophobic void problem arises: the neutral-lipid (triolein) core supplies material to fill the nascent connection, whereas bilayers lack such a reservoir and therefore incur an additional elastic cost associated with tilt to avoid voids [39]. Because direct measurement of the tilt modulus is challenging, we propose that the offset between bilayer and monolayer stalk-formation barriers can be used to experimentally estimate the bilayer tilt modulus—assuming hydration and Gaussian curvature contributions are comparable in both systems—thereby using LD monolayer fusion as a tilt-free reference process.

In cells, lowering the monolayer-fusion barrier by enriching PE biases the LD population toward fewer, larger droplets with a lower surface-to-volume ratio and potentially reduced lipase accessibility—a scenario relevant to hepatic steatosis and adipocyte hypertrophy [10,11]. Conversely, PC-rich shells resist coalescence and stabilize numerous smaller LDs, favoring dynamic turnover. Layering protein factors that mediate LD coalescence (e.g., the CIDE family) on top of the composition-defined baseline will clarify how cells combine biochemical control with intrinsic physicochemical barriers [5]. For LD-mimetic delivery systems [13], our composition–barrier map yields quantitative design rules: PE enrichment trades storage stability for faster coalescence-driven release, whereas PC enrichment enhances shelf stability. Consistent with the curvature-aware model, tuning droplet size within the ~50–200 nm range only weakly affects the rate-determining barrier, so composition is the primary control knob.

In summary, monolayer fusion is the rate-limiting step of protein-free LD coalescence, and DOPE enrichment quantitatively lowers the activation barrier, as extracted from Arrhenius analysis of early-time kinetics. A curvature-aware fusion model captures the composition trend and explains the weak size dependence of the barrier, providing composition-based design rules relevant to LD biology and to engineering LD-mimetic emulsions and delivery systems. Ultimately, changes in lipid composition directly and predictably control the stability and dynamics of lipid emulsions, offering a robust experimental foundation for investigating LD size control and stability in vitro and in vivo and underscoring the value of an integrated experimental–theoretical approach.

## 4. Materials and Methods

### 4.1. Materials

*Phospholipids*: 1,2-dioleoyl-sn-glycero-3-phosphatidylcholine (DOPC) and 1,2-dioleoyl-sn-glycero-3-phosphatidylethanolamine (DOPE) were obtained from Avanti Polar Lipids (Alabaster, AL, USA). *Solvents and reagents*: HPLC chloroform (≥99.8%, Sigma-Aldrich, Saint-Louis, MO, USA) was used to prepare 10 mg/mL lipid stock solutions. Phosphate-buffered saline (PBS; Helicon, Moscow, Russia) was prepared with ultra-pure water (Milli-Q, Direct-Q 3UV, Millipore, Burlington, MA, USA; resistivity ≥ 18.2 MΩ·cm at 25 °C). *Neutral lipid*: Triolein was purified by vacuum distillation using an oil pump (vacuum of about 0.01 mm) according to conventional procedure prior to use [40].

### 4.2. Preparation of Adiposome Emulsions

Adiposome preparation followed [41] with minor adjustments. Chloroform stock solutions of DOPC and DOPE (10 mg/mL) were mixed with triolein in a round-bottom flask to achieve a total phospholipid–triolein molar ratio of 2:5. The total mass of lipid and triolein was 2 mg. Therefore, 0.5 mg of lipid and 1.5 mg of triolein were used. The solvent was removed on a rotary evaporator (40 min at 40 mbar), forming a thin lipid film containing triolein. The film was hydrated with 100 µL of PBS (pH 7.4) and vortexed (10 cycles × 10 s). The crude dispersion was centrifuged (Eppendorf Centrifuge 5804 R, Eppendorf, Hamburg, Germany) at 1000× *g* for 5 min at 4 °C to separate upper and lower fractions. The lower fraction (90 µL, avoiding the top foam with excess oil droplets) was transferred using a capillary pipette (Vertex-GL Gel-Loading Pipette Tip, SSIbio, sterile, Lodi, CA, USA) to a clean tube and centrifuged at 20,000× *g* for 5 min at 4 °C (Eppendorf Centrifuge 5804 R, Eppendorf, Hamburg, Germany). The resulting sediment was discarded, and the supernatant, which was the adiposome suspension, was collected into a clean tube. The volume was adjusted back to 100 µL with PBS. This purification cycle was repeated twice. For DLS measurements, the final adiposome suspension was brought to 600 µL with PBS.

### 4.3. DLS Measurements

Particle size distributions were measured by dynamic light scattering (Zetasizer Nano ZS, Malvern Panalytical, Great Malvern, UK) using backscatter detection at 173°. Measurements accounted for the refractive index of the dispersed lipid phase and the temperature-dependent viscosity of PBS over 10–45 °C. For kinetic series at fixed temperature, data were acquired every 2 min for the first 3 h and every 20 min thereafter for a total of 20–60 h; each time point is the average of 12–15 runs. Data were processed with the Zetasizer Software v8.0. Distributions were generally narrow and monomodal within 30–300 nm. The mean adiposome diameter, *d*, was obtained from both the number-weighted distribution and the intensity-weighted cumulants mean (Z-average), together with the polydispersity index (PDI). The time dependence *d*(*t*) at constant temperature was extracted from both metrics. For every lipid composition and temperature, 3 technical repeats were performed to obtain the average value of *K*.

### 4.4. SAXS Measurements

SAXS experiments were performed at the BioMUR beamline of the Kurchatov Synchrotron Radiation Source (National Research Center “Kurchatov Institute”, Moscow, Russia) [42]. Monochromatic X-rays of wavelength *λ* = 0.145 nm (*E* = 8.58 keV) were used with a sample to detector distance of 2500 mm, covering 0.04 nm−1 < *s* < 1.5 nm^−1^, where *s* = 4π sin(*θ*)/*λ* and 2*θ* is the scattering angle. Fresh DOPC:DOPE adiposomes (35 mol% DOPE) were loaded into quartz capillaries (outer diameter 2 mm; wall thickness 10 µm) and mounted in a thermostatted holder at 35 °C. Two-dimensional patterns were recorded on a PILATUS3 1M detector (Dectris, Baden-Daettwil, Switzerland) over 4 h with 10 min intervals. The two-dimensional scattering pattern of the sample was averaged over the radial direction using the FIT2D program [43]. Buffer scattering was subtracted using PRIMUS [44]. The radius of gyration, *R_g_*, of the lipid droplets was estimated via the Guinier approximation for small-angle scattering, *s* < 1.3/*R_g_*, were *I*(*s*) ≅ *I*(0) exp[−(*sR_g_*)^2^/3] [45]. Adiposome size distributions were reconstructed with the MIXTURES module of ATSAS 3.2.1 suite [46] using a three-component polydisperse sphere model (volume fraction, mean size, and PdI per component).

## Figures and Tables

**Table 1:** Characteristics of lipid droplet emulsions with different lipid compositions of the shell, obtained by the DLS method (average ± s.d., *n* = 10).

Molar Ratio DOPC:DOPE		Hydrodynamic Diameter *D*, nm		PdI
	Number Mean	Intensity Mean	Z-Average	
100:0	62 ± 5	116 ± 22	94 ± 2	0.14 ± 0.01
80:20	69 ± 4	169 ± 43	111 ± 2	0.17 ± 0.01
65:35	61 ± 7	262 ± 42	122 ± 2	0.24 ± 0.01
50:50	69 ± 12	280 ± 58	153 ± 6	0.27 ± 0.03

## Data Availability

The raw data supporting the conclusions of this article will be made available by the authors on request.

## Supplementary Materials

The following supporting information can be downloaded at: https://www.mdpi.com/article/10.3390/ijms262311664/s1. References [47,48,49,50,51] are cited in the supplementary materials.

## Abbreviations

The following abbreviations are used in this manuscript:

LD	Lipid droplet
DOPC	1,2-dioleoyl-sn-glycero-3-phosphatidylcholine
DOPE	1,2-dioleoyl-sn-glycero-3-phosphatidylethanolamine
DLS	Dynamic light scattering
SAXS	Small-angle X-ray scattering
PdI	Polydispersity index
PE	Phosphatidylethanolamine

## References

[1] Tauchi-Sato K., Ozeki S., Houjou T., Taguchi R., Fujimoto T. (2002). The Surface of Lipid Droplets Is a Phospholipid Monolayer with a Unique Fatty Acid Composition. J. Biol. Chem..

[2] Thiam A.R., Farese R.V., Walther T.C. (2013). The Biophysics and Cell Biology of Lipid Droplets. Nat. Rev. Mol. Cell Biol..

[3] Wilfling F., Haas J.T., Walther T.C., Farese R.V. (2014). Lipid Droplet Biogenesis. Curr. Opin. Cell Biol..

[4] Olzmann J.A., Carvalho P. (2019). Dynamics and Functions of Lipid Droplets. Nat. Rev. Mol. Cell Biol..

[5] Gao G., Chen F.-J., Zhou L., Su L., Xu D., Xu L., Li P. (2017). Control of Lipid Droplet Fusion and Growth by CIDE Family Proteins. Biochim. Biophys. Acta (BBA)-Mol. Cell Biol. Lipids.

[6] Gong J., Sun Z., Wu L., Xu W., Schieber N., Xu D., Shui G., Yang H., Parton R.G., Li P. (2011). Fsp27 Promotes Lipid Droplet Growth by Lipid Exchange and Transfer at Lipid Droplet Contact Sites. J. Cell Biol..

[7] Cohen B.-C., Raz C., Shamay A., Argov-Argaman N. (2017). Lipid Droplet Fusion in Mammary Epithelial Cells Is Regulated by Phosphatidylethanolamine Metabolism. J. Mammary Gland. Biol. Neoplasia.

[8] Masedunskas A., Chen Y., Stussman R., Weigert R., Mather I.H. (2017). Kinetics of Milk Lipid Droplet Transport, Growth, and Secretion Revealed by Intravital Imaging: Lipid Droplet Release Is Intermittently Stimulated by Oxytocin. MBoC.

[9] Yang H., Galea A., Sytnyk V., Crossley M. (2012). Controlling the Size of Lipid Droplets: Lipid and Protein Factors. Curr. Opin. Cell Biol..

[10] Cohen B.-C., Shamay A., Argov-Argaman N. (2015). Regulation of Lipid Droplet Size in Mammary Epithelial Cells by Remodeling of Membrane Lipid Composition—A Potential Mechanism. PLoS ONE.

[11] Gluchowski N.L., Becuwe M., Walther T.C., Farese R.V. (2017). Lipid Droplets and Liver Disease: From Basic Biology to Clinical Implications. Nat. Rev. Gastroenterol. Hepatol..

[12] Liang T., Wen D., Chen G., Chan A., Chen Z., Li H., Wang Z., Han X., Jiang L., Zhu J. (2021). Adipocyte-Derived Anticancer Lipid Droplets. Adv. Mater..

[13] Zhao P., Zhao Z., Yu Z., Chen L., Jin Y., Wu J., Ren Z. (2023). Application of Synthetic Lipid Droplets in Metabolic Diseases. Clin. Transl. Med..

[14] Akimov S.A., Molotkovsky R.J., Kuzmin P.I., Galimzyanov T.R., Batishchev O.V. (2020). Continuum Models of Membrane Fusion: Evolution of the Theory. Int. J. Mol. Sci..

[15] Chernomordik L.V., Kozlov M.M. (2008). Mechanics of Membrane Fusion. Nat. Struct. Mol. Biol..

[16] Pavlov R.V., Akimov S.A., Dashinimaev E.B., Bashkirov P.V. (2024). Boosting Lipofection Efficiency Through Enhanced Membrane Fusion Mechanisms. Int. J. Mol. Sci..

[17] Kozlovsky Y., Kozlov M.M. (2002). Stalk Model of Membrane Fusion: Solution of Energy Crisis. Biophys. J..

[18] Ryham R.J., Klotz T.S., Yao L., Cohen F.S. (2016). Calculating Transition Energy Barriers and Characterizing Activation States for Steps of Fusion. Biophys. J..

[19] Borwankar R.P., Lobo L.A., Wasan D.T. (1992). Emulsion Stability—Kinetics of Flocculation and Coalescence. Colloids Surf..

[20] Simovic S., Prestidge C.A. (2004). Nanoparticles of Varying Hydrophobicity at the Emulsion Droplet−Water Interface: Adsorption and Coalescence Stability. Langmuir.

[21] Dinh H.-H.-Q., Santanach-Carreras E., Lalanne-Aulet M., Schmitt V., Panizza P., Lequeux F. (2021). Effect of a Surfactant Mixture on Coalescence Occurring in Concentrated Emulsions: The Hole Nucleation Theory Revisited. Langmuir.

[22] Kabalnov A., Wennerström H. (1996). Macroemulsion Stability: The Oriented Wedge Theory Revisited. Langmuir.

[23] Saito H., Kawagishi A., Tanaka M., Tanimoto T., Okada S., Komatsu H., Handa T. (1999). Coalescence of Lipid Emulsions in Floating and Freeze–Thawing Processes: Examination of the Coalescence Transition State Theory. J. Colloid. Interface Sci..

[24] Molotkovsky R.J., Galimzyanov T.R., Minkevich M.M., Pinigin K.V., Kuzmin P.I., Bashkirov P.V. (2025). Energy Pathway of Lipid Monolayer Fusion: From Droplet Contact to Coalescence. J. Phys. Chem. B.

[25] Wang Y., Zhou X.-M., Ma X., Du Y., Zheng L., Liu P. (2016). Construction of Nanodroplet/Adiposome and Artificial Lipid Droplets. ACS Nano.

[26] Taylor P. (1998). Ostwald Ripening in Emulsions. Adv. Colloid. Interface Sci..

[27] Urbina-Villalba G., Toro-Mendoza J., García-Sucre M. (2005). Calculation of Flocculation and Coalescence Rates for Concentrated Dispersions Using Emulsion Stability Simulations. Langmuir.

[28] Leikin S.L., Kozlov M.M., Chernomordik L.V., Markin V.S., Chizmadzhev Y.A. (1987). Membrane Fusion: Overcoming of the Hydration Barrier and Local Restructuring. J. Theor. Biol..

[29] Kollmitzer B., Heftberger P., Rappolt M., Pabst G. (2013). Monolayer Spontaneous Curvature of Raft-Forming Membrane Lipids. Soft Matter.

[30] Bashkirov P.V., Kuzmin P.I., Vera Lillo J., Frolov V.A. (2022). Molecular Shape Solution for Mesoscopic Remodeling of Cellular Membranes. Annu. Rev. Biophys..

[31] Niebergall L.J., Jacobs R.L., Chaba T., Vance D.E. (2011). Phosphatidylcholine Protects against Steatosis in Mice but Not Non-Alcoholic Steatohepatitis. Biochim. Biophys. Acta (BBA)-Mol. Cell Biol. Lipids.

[32] Aeffner S., Reusch T., Weinhausen B., Salditt T. (2012). Energetics of Stalk Intermediates in Membrane Fusion Are Controlled by Lipid Composition. Proc. Natl. Acad. Sci. USA.

[33] Kalutsky M.A., Galimzyanov T.R., Molotkovsky R.J. (2022). A Model of Lipid Monolayer–Bilayer Fusion of Lipid Droplets and Peroxisomes. Membranes.

[34] Nannette C., Baudry J., Chen A., Song Y., Shglabow A., Bremond N., Démoulin D., Walters J., Weitz D.A., Bibette J. (2024). Thin Adhesive Oil Films Lead to Anomalously Stable Mixtures of Water in Oil. Science.

[35] Pays K., Giermanska-Kahn J., Pouligny B., Bibette J., Leal-Calderon F. (2001). Coalescence in Surfactant-Stabilized Double Emulsions. Langmuir.

[36] Rekvig L., Frenkel D. (2007). Molecular Simulations of Droplet Coalescence in Oil/Water/Surfactant Systems. J. Chem. Phys..

[37] Kozlovsky Y., Efrat A., Siegel D.A., Kozlov M.M. (2004). Stalk Phase Formation: Effects of Dehydration and Saddle Splay Modulus. Biophys. J..

[38] Khattari Z., Köhler S., Xu Y., Aeffner S., Salditt T. (2015). Stalk Formation as a Function of Lipid Composition Studied by X-Ray Reflectivity. Biochim. Biophys. Acta (BBA)-Biomembr..

[39] Kuzmin P.I., Zimmerberg J., Chizmadzhev Y.A., Cohen F.S. (2001). A Quantitative Model for Membrane Fusion Based on Low-Energy Intermediates. Proc. Natl. Acad. Sci. USA.

[40] Armarego W.L.F. (2017). Purification of Laboratory Chemicals.

[41] Zhi Z., Ma X., Zhou C., Mechler A., Zhang S., Liu P. (2022). Protocol for Using Artificial Lipid Droplets to Study the Binding Affinity of Lipid Droplet-Associated Proteins. STAR Protoc..

[42] Peters G.S., Gaponov Y.A., Konarev P.V., Marchenkova M.A., Ilina K.B., Volkov V.V., Pisarevsky Y.V., Kovalchuk M.V. (2022). Upgrade of the BioMUR Beamline at the Kurchatov Synchrotron Radiation Source for Serial Small-Angle X-Ray Scattering Experiments in Solutions. Nucl. Instrum. Methods Phys. Res. Sect. A Accel. Spectrometers Detect. Assoc. Equip..

[43] Hammersley A.P. (2016). *FIT2D*: A Multi-Purpose Data Reduction, Analysis and Visualization Program. J. Appl. Crystallogr..

[44] Konarev P.V., Volkov V.V., Sokolova A.V., Koch M.H.J., Svergun D.I. (2003). *PRIMUS*: A Windows PC-Based System for Small-Angle Scattering Data Analysis. J. Appl. Crystallogr..

[45] Guinier A. (1939). La Diffraction Des Rayons X Aux Très Petits Angles: Application à l’étude de Phénomènes Ultramicroscopiques. Ann. Phys..

[46] Manalastas-Cantos K., Konarev P.V., Hajizadeh N.R., Kikhney A.G., Petoukhov M.V., Molodenskiy D.S., Panjkovich A., Mertens H.D.T., Gruzinov A., Borges C. (2021). *ATSAS 3.0*: Expanded Functionality and New Tools for Small-Angle Scattering Data Analysis. J. Appl. Crystallogr..

[47] Helfrich W. (1973). Elastic Properties of Lipid Bilayers: Theory and Possible Experiments. Z. Für Naturforschung C.

[48] Israelachvili J., Pashley R. (1982). The Hydrophobic Interaction Is Long Range, Decaying Exponentially with Distance. Nature.

[49] Jülicher F., Seifert U. (1994). Shape Equations for Axisymmetric Vesicles: A Clarification. Phys. Rev. E.

[50] Rand R.P., Parsegian V.A. (1989). Hydration Forces between Phospholipid Bilayers. Biochim. Et Biophys. Acta (BBA)-Rev. Biomembr..

[51] Rawicz W., Olbrich K.C., McIntosh T., Needham D., Evans E. (2000). Effect of Chain Length and Unsaturation on Elasticity of Lipid Bilayers. Biophys. J..

