# Supplementary material for: Quantifying the Activation Barrier for Phospholipid Monolayer Fusion Governing Lipid Droplet Coalescence
Source: Int J Mol Sci. 2025 Dec 2;26(23):11664. doi: 10.3390/ijms262311664 (PMC12692553; doi:10.3390/ijms262311664)
Supplement: Supplementary file 1 [file ijms-26-11664-s001.zip › ijms-3992372-supplementary.pdf]

Quantifying the Activation Barrier for Phospholipid Monolayer Fusion Governing Lipid Droplet Coalescence

Supplementary Material

Rodion J. Molotkovsky [1],*, Zaret G. Denieva [2], Ivan N. Senchikhin [2], Ekaterina K. Urodkova [2], Petr V. Konarev [3], Georgy S. Peters [3], Timur R. Galimzyanov [4], Rais V. Pavlov [1] and Pavel V. Bashkirov [1],*.

1 Research Institute for Systems Biology and Medicine (RISBM), Nauchnyi proezd 18, 117246 Moscow, Russia
2 A.N. Frumkin Institute of Physical Chemistry and Electrochemistry, Russian Academy of Sciences, 119071 Moscow, Russia
3 National Research Centre "Kurchatov Institute", Akademika Kurchatova pl. 1, 123182, Moscow, Russia
4 Independent researcher, Munich, Germany
* Correspondence:
RJM: molotkovskiy_ru@sysbiomed.ru;
PVB: pavel.bashkirov@sysbiomed.ru

Small-angle X-ray scattering (SAXS) experiments.

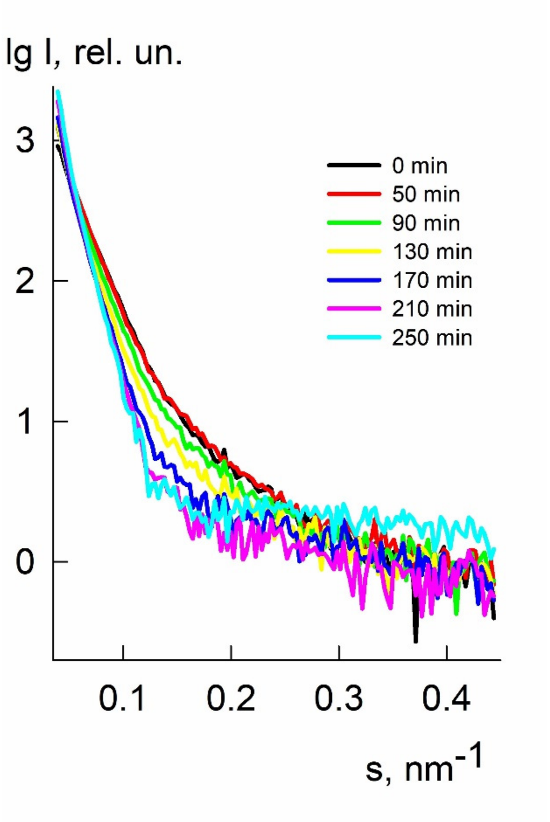

(a)

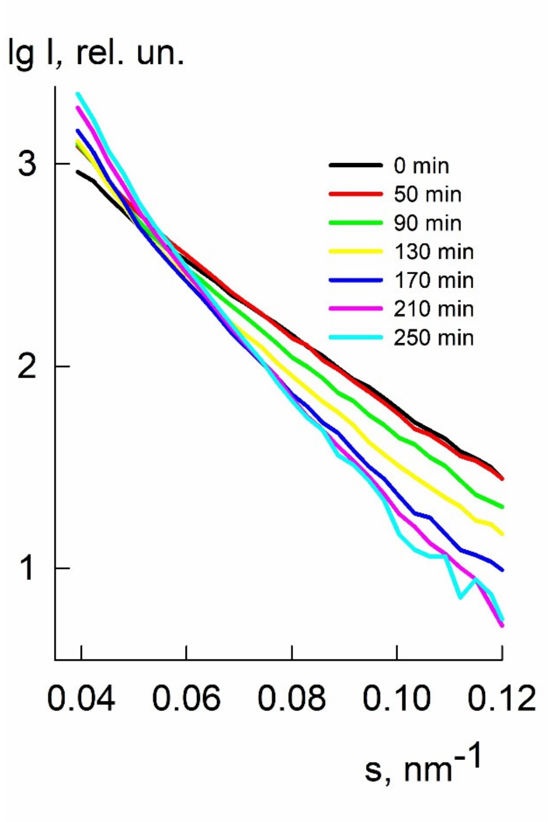

(b)

Figure S1. SAXS profiles of adiposomes (DOPC:DOPE 65:35) collected at 35 °C over 4 h. (a) Full I(q) versus q curves at successive time points. (b) Low-q region highlighting the time-dependent increase in the decay (steeper slope) of the scattering, consistent with progressive droplet growth/coalescence.

Approximation of kinetic data. For each temperature and DOPE fraction, we analyzed diameter time-series $d(t)$, where d is the DLS intensity-weighted cumulants mean (Z-average) at fixed temperature. The sampling interval was 2 min during the first 3h and 20 min thereafter. Raw traces were smoothed with a 4-point moving average filter. The initial diameter d_0 was defined as the mean of the first 10 time points. We then plotted $y(t) = \ln[d_0^3/d(t)^3]$ versus time and the early-time linear regime by ordinary least squares; the cutoff time was selected by maximizing the coefficient of determination (R^2) across candidate truncations. The coalescence rate constant K was taken as the slope of the optimal linear fit. An example illustrating the fit and cutoff selection is shown in Fig. S2.

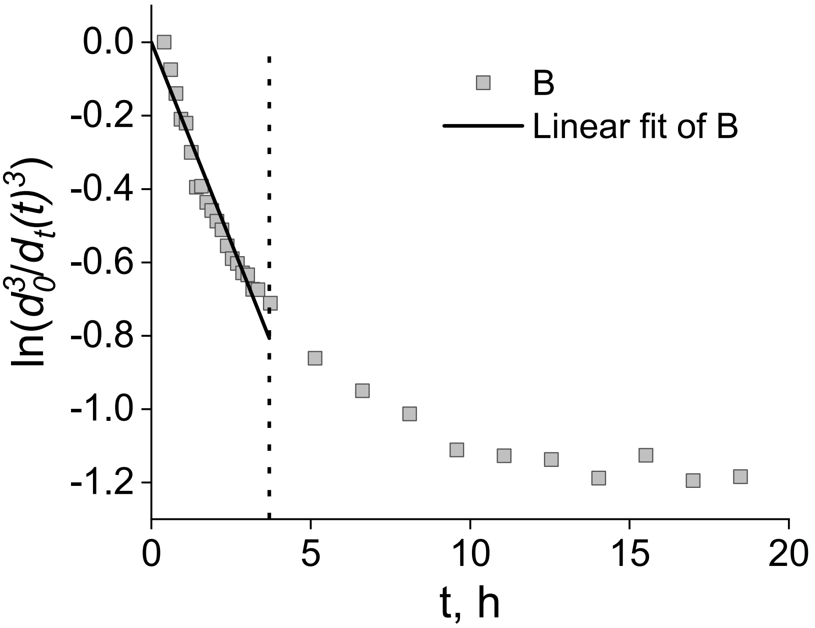

Figure S2. $\ln(d_0^3/d(t)^3)$ versus time (hours) for a data set with 50% DOPE at 30 ˚C. The cutoff is shown as a dashed line and corresponds to a time of 3.7 hours.

A total of 7 data sets were analyzed for both the 35% DOPE case and 50% DOPE case. Values of the coalescence constants with confidence intervals and R^2 values are summarized in Tables S1 and S2.

Table S1. Calculated coalescence constants K, h^{-1}.

T, ˚C	DOPE content	
	35mol%	50mol%
10	0.011±0.002	0.067±0.007
15	0.032±0.004	0.052±0.002
20	0.05±0.005	0.078±0.025
25	0.052±0.007	0.17±0.05
30	0.073±0.011	0.12±0.04
35	0.121±0.013	0.23±0.02
40	0.21±0.05	0.17±0.03

Theoretical modelling. We consider fusion between two apposed spherical monolayers (droplet shells) of equal radius of curvature R_c (Fig. S3). Fusion requires local deformations that bring the monolayers into near contact and nucleate a "stalk"(Molotkovsky et al. 2025). We assume the deformation is confined to circular patches of radius R, with $R << R_c$, and that the two monolayers deform symmetrically; thus, it suffices to analyze a single (lower) monolayer and mirror the solution to the upper one. We place the origin O of a cylindrical coordinate system (r, z) at the center of the circular patch of radius R on the undeformed lower monolayer; z is normal to the monolayer and directed outward from the droplet center, and r is the in-plane radial coordinate. The boundary of the deformed patch is at $r = R$, where the intermonolayer spacing is H_0 (Fig. S3).

Within each patch, we allow for localized, circular hydrophobic defects of radius ρ that arise from partial exposure of LD hydrophobic moiety to water. Defects are positioned symmetrically in the opposing monolayers with center-to-center separation d along z. As d decreases, the effective surface tension at the water/nonpolar interface is reduced, leading to attractive interaction between the defects. When the defects meet (d = 0), a continuous lipid bridge forms between the monolayers – the monolayer stalk – marking the onset of fusion.

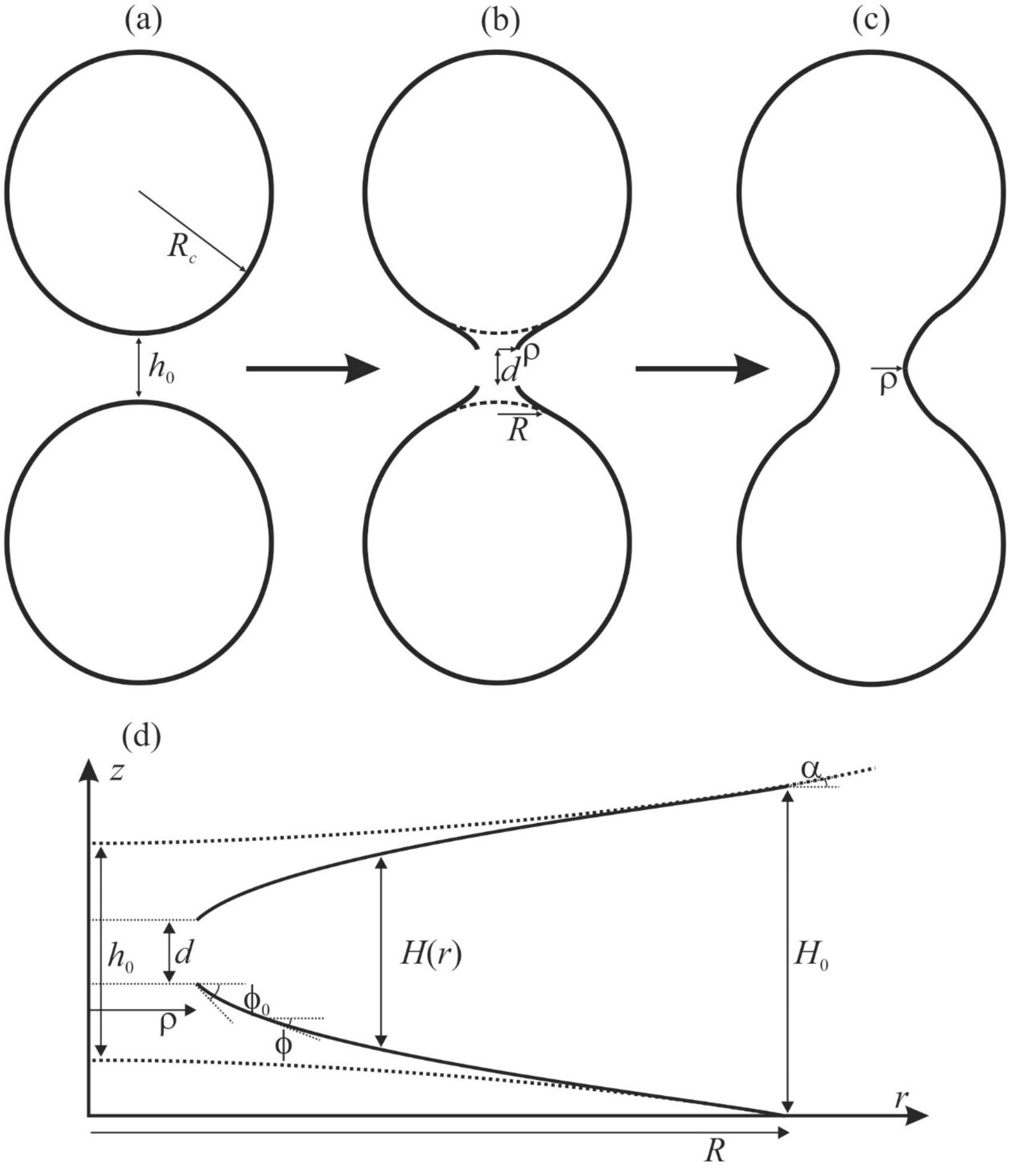

Figure S3. Schematic of the model for monolayer fusion between two apposed spherical vesicles. (a) Initial state. (b) Attraction of opposing hydrophobic defects and stalk nucleation. (c) Stalk expansion. (d) Geometry of hydrophobic defects during stalk formation. Undeformed monolayers are shown as dotted lines. R_c droplet radius; R, radius of the locale deformed spherical segment; h_0 – minimum spacing between the undeformed monolayers; ρ, defect radius before stalk formation and stalk radius thereafter; d center-to-center separation of opposing defects along z; $H(r)$, intermonolayer spacing within the patch, varying from d at the defect rim ($r = \rho$) to H_0 at the patch edge ($r = R$). The monolayer contour is parametrized by the arc length s and tangential angle $\phi(s)$; $\phi(s) = \phi_0$ at $r = \rho$, and $\phi(s) = \alpha$ at $r = R$.

The energy of the system is determined by the shape of the deformed parts of the monolayers. The monolayer is considered as an infinitely thin two-dimensional film with bending rigidity, which corresponds to the Helfrich model (Helfrich 1973). The shape of the monolayer is parameterized by the tangential angle ϕ and the arc length s along the contour. Coordinates $r(s)$ and $z(s)$ depend on s and ϕ as follows:

$$\frac{dr}{ds} = \cos(\phi), \quad \frac{dz}{ds} = -\sin(\phi). \qquad \text{(S1)}$$

Equation (S1) allows us to obtain the function $z(r)$ for a known monolayer shape. The boundary conditions for the function $z(r)$ are as follows:

$$z(R) = 0,$$
$$z(\rho) = \frac{H_0 - d}{2}, \qquad \text{(S2)}$$
$$\left.\frac{dz}{dr}\right|_{r=R} = \frac{R}{R_c} = \sin(\alpha),$$

where $\alpha = \arcsin(R/R_c)$ — tilt of the vesicle monolayer at the border with the deformed segment. The equilibrium shape of the monolayer is found by minimizing the total energy W_t for given values of d and ρ taking into account the boundary conditions (S2).

The total energy of the system W_t is the sum of several components: the elastic energy of the monolayer deformation W_{el}, the hydration repulsion energy W_{hyd}, the energy of hydrophobic defects W_{ph} and the energy associated with surface tension W_s. We also separately consider the elastic energy term associated with the so-called Gaussian curvature; it is denoted as W_G. All energy components are measured from the state of undeformed monolayer segments; thus, the quantity W_t represents the change in the system's energy during the fusion of monolayers.

The change in elastic energy of one monolayer during its deformation is determined in accordance with the Helfrich approximation and is equal to (Jülicher and Seifert 1994)

$$W_{el} = \pi B \int_S \left(\left(\frac{d\phi}{ds} + \frac{\sin(\phi)}{r(s)} - J_s \right)^2 - J_s^2 \right) r(s)\, ds - W_0, \qquad \text{(S3)}$$

where B is monolayer bending modulus, J_s is the monolayer spontaneous curvature, characterizing the averaged geometric form of the lipid molecules, S is integration area form $r = \rho$ to $r = R$, and W_0 is the monolayer elastic energy at the initial state. The value of W_0 is determined by the size of the deformable segment of the monolayer and in the first non-vanishing order of smallness is equal to

$$W_0 = 2\pi B (1 - R_c J_s) \frac{R^2}{R_c^2}. \qquad \text{(S4)}$$

The energy of hydration repulsion is associated with the interaction of oriented water layers at subnanometer distances between monolayers (Rand and Parsegian 1989) and is equal to (Ryham et al. 2016)

$$W_{hyd} = 2\pi \int_S r P_0 \lambda e^{-\frac{H(r)}{\lambda}} \cos^2(\phi)\, ds . \tag{S5}$$

Here $H(r) = H_0 - 2z(r)$ is the distance between, P_0 — the disjoining pressure between flat membranes, λ is the characteristic length of decrease of hydration repulsion, which is of the order of 0.1 – 0.3 nm, and the factor $\cos^2(\phi)$ takes into account tilting of the monolayers relative to each other. Note that formula (S5) does not take into account the initial energy of hydration repulsion; this assumption is justified due to the smallness of the value of λ compared to the distance H_0, equal to several nanometers. Energy W_{ph}, associated with hydrophobic defects is equal to (Israelachvili and Pashley 1982)

$$W_{ph} = 2\pi\rho^2 \sigma_p \left(1 - \exp\left(-\frac{d}{\xi_p}\right)\right), \tag{S6}$$

whew σ_p is the lateral tension at the water/oil interface, and ξ_p is the characteristic length of hydrophobic interactions, equal to 1 nm (Israelachvili and Pashley 1982). The energy associated with the surface tension σ is proportional to the change in the area of the system and is equal to (per monolayer)

$$W_{hyd} = 2\pi\sigma \int_S r(s) ds - \sigma\pi\left(R^2 - \rho^2\right). \tag{S7}$$

Finally, the energy W_G associated with the Gaussian curvature is equal to (Molotkovsky et al. 2025)

$$W_G = 2\pi\kappa\left(\cos(\phi_0) - 1\right), \tag{S8}$$

where κ is the Guassian curvature modulus, and ϕ_0 is the tangential angle at the monolayer boundary with hydrophobic defect. The change in the total energy of the system W_t consists of components (S3–S8) and is equal to

$$W_t = 2\left(W_{el} + W_G + W_s\right) + W_{hyd} + W_{ph} . \tag{S9}$$

Energy (S9) is numerically minimized taking into account the boundary conditions (S2), which allows us to determine the value of W_t as a function of the reaction coordinates d and ρ of the process. Until the moment of formation of a monolayer stalk, energy is minimized with respect to d for each given value of ρ and reaction coordinate is the defect radius ρ. Subsequently, the expansion of the stalk is considered; in this case, the reaction coordinate is the stalk radius ρ, while d is set zero.

Using the calculation algorithm described above, we obtain the energy trajectory of the fusion of monolayer vesicles of different sizes as a dependence of the change in the total energy W_t on the reaction coordinate. For this purpose, it is necessary to determine the elastic and geometric parameters that will be used in the calculations. Thus, a change in the radius of curvature of the vesicle R_c leads to a change in the angle α in the boundary condition (S2), a change in the initial energy of the segment W_0, and also affects the distance between the vesicles. Due to the curvature of the vesicles, the distance between them at the point $r = 0$ will differ from the distance H_0 at the point $r = R$. At the same time, it is the distance at the point r = 0, as the smallest distance, that should remain unchanged when varying the radius of curvature R_c. Therefore, we take the distance between flat monolayers h_0 as the standard of comparison; this distance is assumed to be

5 nm, corresponding to the case of flat monolayers. The distance H_0 at the point $r = R$ is related to it as

$$H_0 = h_0 + 2R_c\left(1-\cos(\alpha)\right) \approx h_0 + \frac{R^2}{R_c}. \quad \text{(S10)}$$

The value of R is assumed to be 10 nm in most calculations.

In addition to vesicles of varying curvature, we also model monolayers of different lipid compositions; we consider compositions with 0, 20, 35 and 50% DOPE. The change in DOPE fraction is modeled as a variation in the elastic properties and hydration repulsion parameters. The dependence of the spontaneous curvature J_s on composition is determined according to the linear approximation.

$$J_s = c_{\text{DOPE}} j_{\text{DOPE}} + \left(1-c_{\text{DOPE}}\right) j_{\text{DOPC}}, \quad \text{(S11)}$$

where c_{DOPE} is the surface fraction of DOPE in the membrane; j_{DOPC} and j_{DOPE} are the spontaneous curvatures of DOPC and DOPE, respectively. The dependence of the bending modulus on the composition is determined according to the formula (Bashkirov et al. 2022)

$$B_{eff} = \frac{B}{1+\dfrac{Baj^2{}_{DOPE}c_{DOPE}\left(1-c_{DOPE}\right)}{k_B T}}, \quad \text{(S12)}$$

where a is he average area of the lipid molecule, which we will henceforth take to be approximately 0.7 nm^2, and B is the bending modulus of DOPC, equal to 10 k_BT (Rawicz et al. 2000). The Gaussian curvature modulus κ is assumed to be related to the modulus B_{eff} according to the relation $-0.33B_{eff}$ (Kozlovsky et al. 2004). The values of hydration repulsion parameters for 0, 20 and 50% DOPE are taken from the experimental data of (Khattari et al. 2015); in case of 20% values for 25% are taken. The ξ_h value for the 35% composition is taken by extrapolation of known data; the P_0 value is taken as the average value. The dependences of the quantities on the lipid composition are summarized in Table S2.

Table S2. Values of parameters P_0 and ξ_h adapted from (Khattari et al. 2015); bending modulus and spontaneous curvature are determined in accordance with formulas (S11) and (S12). Spontaneous curvature of DOPC is taken equal to $j_{DOPC} = -0.09$ nm^{-1}; spontaneous curvature of DOPE $j_{DOPE} = -0.4$ nm^{-1} (Kollmitzer et al. 2013).

Lipid composition	0% DOPE, 100% DOPC	20% DOPE, 80% DOPC	35% DOPE, 65% DOPC	50% DOPE, 50% DOPC
B_{eff}, k_BT	10	8.5	8	7.8
J_s, nm^{-1}	–0.09	–0.15	–0.2	–0.25
P_0, k_BT/nm^3	360	280	380	485
ξ_h, nm	0.28	0.275	0.22	0.195

The remaining parameters are assumed to be independent on the composition. The membrane surface tension is set to $\sigma = 0.04$ mN/m; the hydrophobic interaction length is assumed to be $\xi_p \approx$ 1 nm; the water/oil tension is assumed to be $\sigma_p \approx 40$ mN/m.

REFERENCES

Helfrich, W. 1973. “Elastic Properties of Lipid Bilayers: Theory and Possible Experiments.” *Zeitschrift für Naturforschung C* 28(11–12): 693–703. doi:10.1515/znc-1973-11-1209.

Israelachvili, Jacob, and Richard Pashley. 1982. “The Hydrophobic Interaction Is Long Range, Decaying Exponentially with Distance.” *Nature* 300(5890): 341–42. doi:10.1038/300341a0.

Jülicher, Frank, and Udo Seifert. 1994. “Shape Equations for Axisymmetric Vesicles: A Clarification.” *Physical Review E* 49(5): 4728–31. doi:10.1103/PhysRevE.49.4728.

Khattari, Ziad, Sebastian Köhler, Yihui Xu, Sebastian Aeffner, and Tim Salditt. 2015. “Stalk Formation as a Function of Lipid Composition Studied by X-Ray Reflectivity.” *Biochimica et Biophysica Acta (BBA) - Biomembranes* 1848(1): 41–50. doi:10.1016/j.bbamem.2014.08.010.

Kollmitzer, Benjamin, Peter Heftberger, Michael Rappolt, and Georg Pabst. 2013. “Monolayer Spontaneous Curvature of Raft-Forming Membrane Lipids.” *Soft Matter* 9(45): 10877. doi:10.1039/c3sm51829a.

Kozlovsky, Yonathan, Avishay Efrat, David A. Siegel, and Michael M. Kozlov. 2004. “Stalk Phase Formation: Effects of Dehydration and Saddle Splay Modulus.” *Biophysical Journal* 87(4): 2508–21. doi:10.1529/biophysj.103.038075.

Molotkovsky, Rodion J., Timur R. Galimzyanov, Mariya M. Minkevich, Konstantin V. Pinigin, Peter I. Kuzmin, and Pavel V. Bashkirov. 2025. “Energy Pathway of Lipid Monolayer Fusion: From Droplet Contact to Coalescence.” *The Journal of Physical Chemistry B*: acs.jpcb.5c02054. doi:10.1021/acs.jpcb.5c02054.

Rand, R.P., and V.A. Parsegian. 1989. “Hydration Forces between Phospholipid Bilayers.” *Biochimica et Biophysica Acta (BBA) - Reviews on Biomembranes* 988(3): 351–76. doi:10.1016/0304-4157(89)90010-5.

Rawicz, W., K.C. Olbrich, T. McIntosh, D. Needham, and E. Evans. 2000. “Effect of Chain Length and Unsaturation on Elasticity of Lipid Bilayers.” *Biophysical Journal* 79(1): 328–39. doi:10.1016/S0006-3495(00)76295-3.

Ryham, Rolf J., Thomas S. Klotz, Lihan Yao, and Fredric S. Cohen. 2016. “Calculating Transition Energy Barriers and Characterizing Activation States for Steps of Fusion.” *Biophysical Journal* 110(5): 1110–24. doi:10.1016/j.bpj.2016.01.013.
